# Topics of Public Interest

**Published:** 1910-01

**Authors:** 


### TOPICS OF PUBLIC INTEREST.

### Red Cross Stamps Popular.

OVER 8,000,000 Red Cross Christmas stamps have been sold to December 20, 1909, in New York State. This statement has just been issued by the National Red Cross and the State Charities Aid Association, who have this sale in charge in this state.

USE OF FUNDS.

The opportunity to raise a substantial fund for local anti-tuberculosis work through the sale of the Red Cross "stickers" has been grasped with great enthusiasm by nearly every city, town and village in the state of any size. The fact that four-fifths of the net proceeds go for this purpose has been a great incentive to local effort. Various plans for using the funds have been announced. Some cities will provide a visiting nurse fund, others are interested in establishing a day and night camp, others wish to start hospitals or dispensaries, and many are just awakening and wish to inaugurate a thorough educational campaign. In Brooklyn, an unusual use of the funds will be made. Two ferry-boat day camps are to be supported by the proceeds of the sale.

UNIQUE METHODS.

In nearly every city in New York State today, there are to be found in the hotel corridors, in the streets and post offices, girls in the uniform of the Red Cross nurse selling the tiny messengers of good-will bearing the familiar Red Cross.

Many innovations in methods of disposing of these stamps have been instituted by various localities. In Buffalo the city has been divided into districts and members of the Association for the Relief and Control of Tuberculosis called up all subscribers informing them of the sale of the stamps. School prizes for the school selling the largest amount of stamps have been announced, the money being raised by bridge parties and other social affairs.

In Rochester, Albany and Binghamton the bill posting firms are coöperating and posting hundreds of sheets announcing the sale which has been particularly successful in Binghamton. The committee there disposed of 10,000 in bulk to one firm. The backs of hundreds of thousands of street car transfers are donated for free advertising. Last year this committee raised $1,500, and is planning on no less than $3,000 as a result of this year's sale. The committee in Troy is canvassing the school teachers by permission of the Board of Education, and expects to increase the sales largely through the school children. The first day of the distribution in Utica resulted in a sale of 21,000 stamps.

In Batavia and many other towns every merchant has the stamps on sale. There is very generous coöperation from church societies, Y. M. C. A., fraternal orders, life insurance companies, Y. W. C. A., and the newspapers are giving extensive notices, and in many instances special editorials. In Newburgh striking cartoons are being published. Kingston has publicly given thanks for results of last year's sale and has issued stirring appeals for large returns. The Geneva tuberculosis committee has organized the whole of Ontario County under the supervision of a paid secretary.

WHERE THE STAMPS ARE SOLD.

The list of the cities and towns that are regularly organised for the sale of the Christmas stamps under the Red Cross Society and the State Charities Aid Association, includes besides the places mentioned, New York city, Cohoes, Elmira, Rome, Schenectady, Ballston, Middletown and other cities and villages numbering over 70.

---

**The Treatment of Diseases in Senility.**

I. L. Nascher of New York, says that the neglected old methods of treatment are many of them useful in treating the diseases of senility. Processes that are pathological in maturity are physiological in senility. Efforts must be made to retard these senile processes. The harmonious relation of the altered functions must be maintained. In senility the heart, when its action is increased, hastens its own decay. Toxemia from decomposing food waste which has not been voided owing to lack of peristaltic activity must be combatted and the resulting weakness overcome. Incidental complications of disease will cause loss of life if not combatted. Vasoconstrictor cardiac stimulants given for weak heart without regard to the condition of the cerebral vessels may cause apoplexy. Two main causes of death are exhaustion or paralysis of the heart and asthenia. The author analyses the effects of the cardiac tonics, and deprecates the use in the aged of those that increase arterial tension. Asthenia may be combatted by diet and abstinence from fatigue. Strychnia and phosphorus are excellent tonics for this condition.—*Medical Record,* December 11, 1909.

---

Gunner—And now comes a professor who declares that fruit is just as healthy with the skin on as it is peeled.

Guyer—H'm! I'd like to see somebody start him on a diet of pineapples.—*Chicago News.*

